# Emotion Processing in Schizophrenia: Insights From a Brain Imaging Study Comparing Patients, Siblings, and Healthy Controls

**DOI:** 10.1002/hbm.70437

**Published:** 2026-02-18

**Authors:** Anna M. Fiorito, Ayman Kheireddine, Hyemin Han, Colas Morel‐Prieur, Nicolas Oriol, Fabien C. Schneider, Guillaume Sescousse, Eric Fakra

**Affiliations:** ^1^ Université Claude Bernard Lyon 1, CNRS, INSERM Centre de Recherche en Neurosciences de Lyon CRNL U1028 UMR5292, PSYR2 Bron France; ^2^ Centre Hospitalier Le Vinatier Bron France; ^3^ Department of Neurosciences “Rita Levi Montalcini” University of Turin Turin Italy; ^4^ Department of Psychiatry University Hospital of Saint‐Etienne Saint‐Etienne France; ^5^ Educational Psychology Program University of Alabama Tuscaloosa Alabama USA; ^6^ Université Jean Monnet Saint‐Etienne, CHU Saint‐Etienne, Unité de Recherche TAPE Saint‐Etienne France

**Keywords:** connectivity, emotion, endophenotype, fMRI, schizophrenia, siblings

## Abstract

Abnormal facial emotion recognition has been proposed as a potential endophenotype for schizophrenia, as both patients with schizophrenia and their healthy relatives show difficulties in recognizing negative facial emotions. In psychiatric disorders, brain functioning is considered highly informative for endophenotype research. However, recent studies have raised questions about our current understanding of the neural correlates of emotion recognition across the schizophrenia spectrum, pointing out two major limitations in previous research. First, individual fMRI studies and meta‐analyses have predominantly used neutral stimuli as a comparator in emotional tasks. Yet, recent evidence indicates that neutral stimuli are not perceived as truly neutral by patients with schizophrenia or their first‐degree relatives, thereby calling into question the interpretability of earlier findings. Second, few studies have explored brain connectivity in response to negative emotional faces in healthy relatives of patients, even though emotions are processed by a complex network of interconnected brain structures. This study aims to investigate the neural mechanisms underlying emotion processing in patients with schizophrenia and their siblings, focusing on brain activation and functional connectivity during the perception of negatively valenced facial expressions. We employed a more ecologically valid task compared with previous studies, incorporating emotional faces within an emotionally charged context. By employing a well‐matched control condition and examining connectivity patterns within emotion‐processing networks, we seek to address the limitations of prior research. 118 participants (37 patients with schizophrenia, 39 siblings, and 42 healthy controls) underwent functional magnetic resonance imaging (fMRI) scanning while performing an emotional task involving negatively valenced faces and control conditions. Behavioral performance was assessed using the Balanced Integration Score (BIS) to evaluate speed‐accuracy tradeoffs. fMRI data were analyzed for brain activation, using a complementary approach based on frequentist and Bayesian statistics, as well as for functional connectivity, using generalized psychophysiological interaction (gPPI) analysis with the right and left amygdala as seed regions. Behavioral analyses revealed significant group differences, with both patients and their siblings displaying lower BIS scores compared with controls. Exploratory between‐group fMRI analyses revealed that, compared with controls, siblings exhibited decreased brain responses to negative faces in a cluster encompassing the right superior temporal gyrus and the right postcentral gyrus. Bayesian analyses showed that, as compared with controls, patients seem to display similarly impaired activation in the right postcentral gyrus. In most of the rest of the brain, including the amygdala, Bayesian analyses indicated an absence of group differences for both patients and siblings compared with controls. Functional connectivity analyses revealed that siblings had stronger task‐related connectivity between the right amygdala and the right cuneus compared with controls. Our findings provide evidence that while the amygdala appears to respond to negative faces similarly among controls, siblings, and patients, an endophenotypic pattern may be present in the right postcentral gyrus. Furthermore, these results suggest that activation‐based analyses may not fully capture the neural abnormalities associated with emotion processing in relatives of patients with schizophrenia. We hypothesize that the increased connectivity between the right amygdala and the right cuneus may reflect a compensatory mechanism in siblings.

**Clinical Registration:**

https://clinicaltrials.gov/study/NCT02834208?term=schizoimagen&rank=1

## Graphical abstract

This fMRI study investigates emotion processing as an endophenotype of schizophrenia by examining patients, siblings, and healthy controls. Using both frequentist and Bayesian voxelwise analyses, we identified a potential endophenotypic marker of dysfunctional emotion processing in the right postcentral gyrus, accompanied by putatively compensatory right amygdala–right cuneus connectivity in siblings.

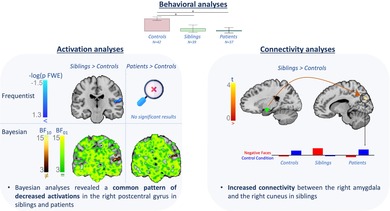

## 1 Introduction

Schizophrenia is a complex psychiatric disorder that affects around 1% of the population worldwide. A core feature of the disorder is impairment in emotion processing, in particular poorer emotion recognition performance. Indeed, numerous studies have shown that patients with schizophrenia have deficits in emotion perception, especially in facial emotion processing (Chan et al. 2010; Kohler et al. 2010). These impairments are especially pronounced when processing negatively valenced facial expressions (Gao et al. 2021), as illustrated by slower reaction times, reduced accuracy, and specific misclassification for these stimuli—for instance, confusing anger with fear or disgust (Bae et al. 2024; Lee et al. 2022). These deficits are clinically significant, as they are associated with more severe negative symptoms and poorer functional outcomes, including social withdrawal and reduced autonomy (Chen et al. 2012; Horan et al. 2012; Irani et al. 2012).

Interestingly, meta‐analytical evidence suggests that first‐degree relatives of individuals with schizophrenia also have difficulties in recognizing facial expressions, particularly those conveying negative emotions (Martin et al. 2020). These findings support the hypothesis that deficits in emotion processing could serve as a potential endophenotype of schizophrenia (Gur et al. 2007)—a measurable component on the pathway from the disease to its genetic basis, which is also present in healthy first‐degree relatives of patients, although less readily observable (Gottesman and Gould 2003).

Neuroimaging measures are particularly valuable in this context, as putative biological endophenotypes are closer to the pathophysiology of the disorder (Glahn et al. 2007; Gur et al. 2007; Hariri and Weinberger 2003). However, whether brain correlates of emotion processing qualify as relevant endophenotypes of schizophrenia remains an open question. Two primary limitations may have hindered progress in this area.

First, in order to specifically examine brain activity related to emotional processing while controlling for visual confounds, functional magnetic resonance imaging (fMRI) studies have traditionally contrasted emotional stimuli with neutral ones. The underlying assumption is that emotional and neutral faces are visually similar, differing primarily in their emotional valence, such that any differences in brain activation can be attributed to emotional processing rather than perceptual properties. However, recent evidence has shown that stimuli labeled as “neutral” for healthy individuals are not perceived as neutral in patients with schizophrenia. This has been corroborated by a recent meta‐analysis reporting that brain activations in response to neutral stimuli differ between patients with schizophrenia and healthy controls (Dugré et al. 2019), with patients showing hyperactivity in the limbic system. Differences were also found between first‐degree relatives of patients with schizophrenia and healthy controls (Fiorito et al. 2024), with relatives exhibiting hypoactivity in limbic areas. As a result, it has been suggested that the hypoactivation in response to emotional stimuli in patients with schizophrenia could be at least partially explained by hyperactivation to neutral stimuli (Anticevic et al. 2012; Dugré et al. 2019), thus calling into question our present comprehension of brain correlates of emotion processing in schizophrenia.

Second, it has been argued that coordinated activity across neural networks, rather than isolated activity within specific regions, is more reflective of complex behaviors like emotion processing (Meehan and Bressler 2012). To investigate emotional face processing more effectively, techniques capable of quantifying the direction and strength of connectivity between brain regions, such as psychophysiological interaction (PPI) analysis, are thus crucial (Gard et al. 2018). Notably, functional connectivity during emotion processing seems to be impaired in schizophrenia (Comte et al. 2017; Potvin et al. 2017). Furthermore, a handful of studies investigating brain connectivity during emotion processing have also observed differences in first‐degree relatives (Cao et al. 2016; Diwadkar et al. 2012; Quarto et al. 2018).

Given these considerations, the current study aimed to investigate the neural mechanisms underlying emotion processing in patients with schizophrenia, their unaffected biological siblings (i.e., first‐degree relatives of the included patients), and healthy controls, addressing the limitations identified in previous research. First, we examined brain activation during the processing of negatively valenced faces—that trigger the most severe behavioral deficits in both patients and siblings—by contrasting them with a non‐emotional control condition (i.e., arrow direction judgment) that was visually engaging and matched in terms of cognitive and motor demands. Second, we focused on functional connectivity, acknowledging that emotion processing is governed by a distributed network of interconnected brain structures. Using generalized psychophysiological interaction (gPPI) analysis with the left and right amygdala as seed regions, we searched for disruptions of coordinated activity within emotion processing networks, which could serve as a more sensitive and specific neural marker for schizophrenia.

## 2 Materials and Methods

### 2.1 Participants

A total of 122 participants took part in this study (40 patients with schizophrenia, 40 siblings of the included patients, and 42 healthy controls). Patients had a diagnosis of schizophrenia, established according to the Diagnostic and Statistical Manual of Mental Disorders (DSM‐5) and confirmed via a psychiatric interview (i.e., Mini‐International Neuropsychiatric Interview, MINI; Sheehan et al. 1998). Patients were regularly monitored in an outpatient hospital setting and were not currently hospitalized. Additionally, the type and dosage of their pharmacological treatment had remained stable for the past 6 months. Importantly, individuals with a history of substance abuse in the past year and those meeting the criteria for treatment‐resistant schizophrenia were excluded, ensuring a clinically stable and relatively homogeneous sample. Siblings (i.e., brothers and sisters) of patients with schizophrenia were considered clinically unaffected: they had no current or past psychiatric diagnoses, as verified using the MINI interview, and did not take psychotropic treatments. Finally, healthy controls had no personal or family history of psychiatric disorder and no psychotropic treatments.

This study was conducted following the principles of the Declaration of Helsinki. Approval was obtained from the local ethics committee (Comité de protection des personnes [CPP] Sud‐Est III, Saint‐Etienne). Each participant gave informed written consent before entering the study.

Three participants (2 patients and 1 sibling) were excluded from the analysis as they exited the MRI scanner before completing the scanning session, resulting in incomplete data acquisition. Furthermore, we excluded data from 1 patient due to excessive head motion during the anatomical scan. Thus, the final sample comprised 37 patients with schizophrenia, 39 siblings of the included patients, and 42 healthy controls.

### 2.2 Task

**FIGURE 1:**
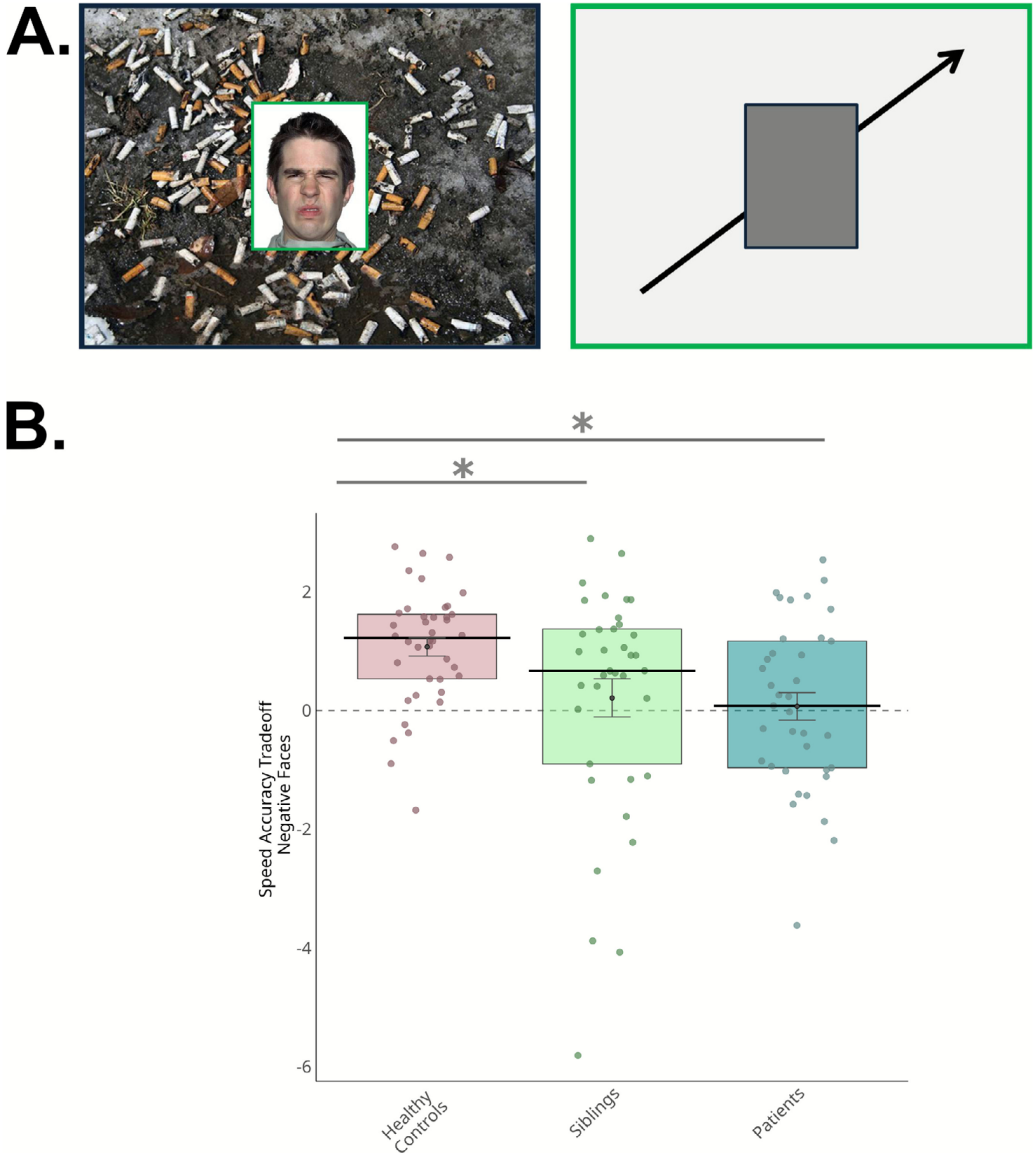
Results of behavioral analyses. Examples of task stimuli illustrating Negative Faces condition and Control Condition are shown in (A). Boxplot (B) illustrates the Speed Accuracy Tradeoff scores (measured by the Balanced Integration Score, BIS) for the Negative Faces condition across the three groups: Healthy controls, siblings, and patients with schizophrenia. We observed an endophenotypic pattern, with siblings exhibiting BIS scores that were intermediate between those of patients and healthy controls. Each box represents the interquartile range, with the median indicated by a black line. The black dot represents the mean, with grey error bars indicating the standard error of the mean. Individual data points are overlaid to show participant‐level variability within each group.

We employed an emotional task that was previously developed in our lab and was proven to be efficient for investigating the neural correlates of emotional processing: the variable attention and congruency task (VAAT, Comte et al. 2016). A detailed description of the task is provided elsewhere (Comte et al. 2016); briefly, the task comprises 4 sessions, and task stimuli were composed of two images: an emotional face in the central part and an emotional scene in the background. Face and scene images varied in their emotional valence (positive or negative), so that they could be congruent or incongruent. In each trial, participants had to focus their attention on the part of the image highlighted in green (either the face or the scene) and report if it depicted a positive or negative emotion using a two‐button response box. The stimulus remained displayed on the screen for 3 s, during which the participant had to answer. Moreover, in a control condition, participants had to determine whether an oblique arrow appearing either in the central part of the image or in the background was pointing to the left or right direction (see Figure 1A for an example of task stimuli).

Unlike traditional tasks used to study emotional processing in schizophrenia, which typically involve the isolated presentation of faces displaying different emotions, this task offers a more ecologically valid approach by integrating both facial expressions and contextual emotional cues. This method more closely mirrors real‐life situations, where we not only interpret emotions from facial expressions but also from the emotional context in which interactions occur.

In the context of the present study, we focused on conditions that more closely replicate previous literature, investigating behavioral and brain responses when participants focused on negatively valenced faces in the absence of emotional incongruency (i.e., where both the face and background scene expressed negative emotional valence), referred to as “Negative Faces” (see Figure 1A for an example of task stimuli). While positive emotional stimuli were included to meet task demands (i.e., determine if a stimulus was positive or negative) and to span the whole range of emotional valence, they were not included in the contrast due to the less consistent impairments observed across clinical and at‐risk populations (e.g., Bae et al. 2024) and the greater variability in associated neural responses (e.g., Kosaka et al. 2002; Li et al. 2012; Pulkkinen et al. 2015).

### 2.3 Behavioral Analyses

Statistical analyses were performed with Matlab (version R2020b) and R (version 4.3.1).

While traditional behavioral analyses often treat accuracy and reaction time as independent variables, this approach can obscure the interplay between speed and performance, particularly in tasks where a tradeoff exists. Thus, to assess the speed–accuracy tradeoff in our study, we utilized the Balanced Integration Score (BIS, Liesefeld and Janczyk 2019). This metric is designed to integrate both accuracy and reaction time with equal weight into a single measure. It corresponds to the difference between standardized accuracy and standardized reaction time (RT) of correct answers:

$${\mathrm{BIS}}_{i,j}=Z_{\text{Accurac}y_{i,j}}-Z_{RT_{i,j}}\text{with}\;Z_{x_{i,j}}=\frac{x_{i,j}-\bar{x}}{SD_{x}}$$

where *i* is the participant and *j* is the condition. The standardized scores (*z*) are calculated across all subjects and conditions. Since higher accuracy and lower reaction times indicate better performance in a task, a higher BIS indicates a better overall task performance.

Using *ezANOVA* function in R, we ran a one‐way analysis of variance (ANOVA) with one within‐group factor (BIS score for negative faces) and one between‐subject factor (group, with 3 levels: patients with schizophrenia, siblings, healthy controls). For significant results, we performed post hoc pairwise *t*‐tests using Tukey's HSD (honestly significant difference) procedure.

### 2.4 fMRI Data Acquisition

Functional MRI data were acquired on a 3T Siemens MAGNETOM Prisma scanner at the IRMAS Center (Saint‐Etienne Hospital Center, France) using a T2*‐weighted echo‐planar imaging (EPI) sequence. The protocol parameters were as follows: repetition time (TR) = 2.32 s, echo time (TE) = 30 ms, flip angle = 90°, field of view (FOV) = 220. Each volume consisted of 45 slices with a slice thickness of 3 mm, resulting in a voxel size of 3 × 3 × 3 mm^3^.

Structural images were acquired using a T1‐weighted magnetization‐prepared rapid gradient‐echo (MPRAGE) sequence, providing high‐resolution anatomical images. The parameters for the structural scan were as follows: repetition time (TR) = 2.8 s, echo time (TE) = 2.27 ms, inversion time (TI) = 1100 ms, flip angle = 8°, field of view (FOV) = 230, 256 slices, and voxel size of 1 × 1 × 1 mm^3^.

### 2.5 fMRI Preprocessing

The fMRI data were converted to BIDS format and preprocessed using the fMRIPrep pipeline (version 23.0.1; Esteban et al. 2020, 2019). This automatic workflow performs minimal preprocessing on both anatomical and functional data.

For the anatomical preprocessing, the T1‐weighted images were corrected for intensity non‐uniformity, skull‐stripped, and segmented into cerebrospinal fluid, white matter, and gray matter. Spatial normalization to MNI standard space (MNI152NLin2009cAsym) was performed using nonlinear registration.

For the functional preprocessing, the 4 blood‐oxygen‐level‐dependent (BOLD) runs underwent slice‐timing correction and were realigned for head motion correction. Field distortion correction was applied using B0 field maps, which were estimated from the phase‐drift map of two consecutive Gradient Echo acquisitions and aligned to the Echo‐planar imaging (EPI) reference image. The corrected images were then co‐registered to the T1‐weighted reference, followed by spatial normalization to MNI space (using voxel dimensions of 2 × 2 × 2 mm^3^). Finally, functional images preprocessed with fMRIPrep were smoothed with a 6 mm full‐width at half‐maximum (*FWHM*) Gaussian kernel.

### 2.6 fMRI Statistical Analysis

#### 2.6.1 Functional Activity

The fMRI data were analyzed using statistical parametric mapping (SPM12; Wellcome Center for Neuroimaging, London, UK; https://www.fil.ion.ucl.ac.uk/spm) implemented in MATLAB (version R2020b) on a Unix computer. Part of the analyses was performed with bidspm (v3.1.0dev; https://github.com/cpp‐lln‐lab/bidspm; Van Audenhaege et al. 2024).

At the subject level, we performed a mass univariate analysis with a linear regression (General Linear Model, GLM) at each voxel of the brain, using generalized least squares. Image intensity scaling was done run‐wide before statistical modeling such that the mean image would have a mean intracerebral intensity of 100.

We modeled the fMRI data using an event‐related design, with regressors entered into the run‐specific design matrix. Each task condition consisted of 32 repetitions, represented by a separate regressor of interest, enabling modeling of the entire task. This design accounted for the combinations of emotional valence (negative or positive), attention (faces or scenes), and congruency (congruent or incongruent scene‐face valence), thus comprising 2 × 2 × 2 = 8 emotion regressors. Additionally, the control condition consisted of 64 repetitions and was modeled as a separate regressor. All regressors were convolved with a canonical hemodynamic response function (HRF). Given the short duration of response times, each trial was modeled as an event, thus considering the duration of neural activity as an impulse (i.e., zero duration).

For the denoising strategy, we followed guidelines for task‐based fMRI (Mascali et al. 2021) and included several nuisance covariates in our GLM: 24 movement regressors parameters (including 6‐realignment parameters, their temporal derivatives, and squared terms) to control for movement, and anatomical component correction strategy (aCompCor) introduced by Behzadi et al. (2007), to control for physiological noise. To prevent data overfitting, we retained a fixed number of principal components, 5 for each tissue type (white matter and cerebrospinal fluid). Data were also high‐pass filtered (128 s) to remove low‐frequency signals, and a FAST model was applied to adjust for serial correlations in the data.

For each participant, we generated a first‐level contrast modeling brain responses to negative faces in the context of a congruent negative background scene compared with the control condition (Negative Faces > Control Condition).

The first‐level contrasts were entered into second‐level analyses. Within‐group activations were examined using one‐sample *t*‐tests. Between‐group differences were assessed using pairwise two‐sample *t*‐tests (patients versus healthy controls; siblings versus healthy controls; patients versus siblings). Results were thresholded using a cluster‐defining threshold of *p*_voxel_ < 0.001 and a familywise error (FWE) corrected cluster significance threshold of *p*_cluster_ < 0.05. Because siblings of patients are themselves healthy and without psychiatric diagnoses, only modest neural differences compared with healthy controls with no familial risk for schizophrenia are anticipated. To better capture these subtle effects, we conducted exploratory analyses using a more liberal cluster‐defining threshold of *p*_voxel_ < 0.005 which increases sensitivity to smaller effect sizes, while maintaining an FWE‐corrected cluster significance threshold of *p*_cluster_ < 0.05.

An explicit mask of grey and white matter was applied in SPM12 to restrict analyses to voxels within the brain.

To complement classical second‐level frequentist analyses, we applied a Bayesian framework to quantify the evidence supporting the presence or absence of group differences. Given that siblings represent a clinically unaffected group expected to show subtle deviations in neural responses, a Bayesian approach provides a principled way to quantify the strength of evidence in favor of either hypothesis, thereby enhancing the interpretability of null or near‐threshold effects. Specifically, we employed the BayesFactorFMRI toolbox (Han 2020, 2021), a tool developed with R and Python code to perform Bayesian voxel‐wise analysis of T‐maps at the second level. This toolbox was customized (by HH) to conduct two‐sample *t*‐tests for pairwise group comparisons, producing a map of Bayes factors (BFs) for each voxel. The BF is the ratio of the likelihood of the data under the null hypothesis (H_0_, hypothesizing the absence of meaningful group differences) and the likelihood of the same data under the alternative hypothesis (H_1_, hypothesizing a meaningful group difference). BF_01_ quantifies the evidence supporting H_0_ relative to H_1_, while BF_10_ (= 1/BF_01_) quantifies the evidence supporting H_1_ relative to H_0_. Conventionally a BF_01_ (or BF_10_) that exceeds the threshold of 3 represents moderate evidence in favor of H_0_ (or H_1_), while a BF between 10 and 30 represents strong evidence (Jeffreys 1998). Results are thus reported at a threshold of BF > 3. It should be noted that BayesFactorFMRI adjusts the prior probability distribution to address the problem of multiple comparisons. Nonetheless, the main results concerning the presence of group differences were derived from classical frequentist whole‐brain analyses with stringent multiple comparison corrections.

#### 2.6.2 Functional Connectivity

Generalized psychophysiological interaction (gPPI, McLaren et al. 2012) was employed to investigate whether the pattern of functional connectivity differed between groups. Given the key role of the amygdala in emotion processing, the left and right amygdala were used as seeds in two separate gPPI analyses. To define the seed mask, we created a 5 mm sphere centered on the amygdala, using the peak coordinates of the one‐sample activations across all groups. Within the seed region, the first eigenvariate of the BOLD time series was extracted and deconvolved with the canonical HRF to generate the physiological regressor. Then, PPI regressors were created as the product of the seed time course and task regressors. We ran a first‐level GLM with the physiological regressor of the seed region, the psychological regressors of the task, and the PPI regressor, as well as all the nuisance variables (24 movement regressors, 5 CompCor for the white matter and 5 CompCor for cerebrospinal fluid). We followed the same procedure as for activation analyses, and we created a first‐level contrast (Negative Faces > Control Condition). Finally, we applied the same statistical approach and thresholding strategy as in the activation analyses: within‐group effects were assessed using one‐sample *t*‐tests, and between‐group differences were examined using pairwise two‐sample *t*‐tests. Results were thresholded using a cluster‐defining threshold of *p*_voxel_ < 0.001 and FWE‐corrected *p*_cluster_ < 0.05. Exploratory analyses were additionally performed for the siblings versus healthy controls contrast to increase sensitivity to subtle group differences (using *p*_voxel_ < 0.005 and FWE‐corrected *p*_cluster_ < 0.05).

## 3 Results

### 3.1 Participants Demographic Characteristics

The groups did not significantly differ in age. However, there was a significant difference in years of education between patients and healthy controls (mean difference = −1.6 years, 95% CI [−2.9, −0.3], *p* = 0.01). Additionally, the sex distribution of siblings differed significantly from that of patients (mean difference = −0.48, 95% CI [−0.73, −0.23], *p* < 0.001) and healthy controls (mean difference = −0.26, 95% CI [−0.50, −0.02], *p* = 0.03). See Table 1 for participants' demographic characteristics.

**TABLE 1:** Participant's demographic characteristics.

	Healthy controls	Siblings	Patients with schizophrenia
Number	42	39	37
Age (years); SD	36.5 ± 8.9	34.7 ± 9.6	35.1 ± 8.0
Sex (M/F)	25/17	13/26	30/7
Education (years); SD	13.1 ± 1.5	18.9 ± 2.9	11.5 ± 2.8
Chlorpromazine equivalent (mg/day; SD)	NA	NA	541.9 ± 450.1

### 3.2 Behavioral Analyses

One‐way ANOVA revealed a significant main effect of the group on the speed‐accuracy tradeoff (*F* = 5.192, *p* = 0.007). Post hoc comparisons using the Tukey HSD test indicated that siblings had a BIS score significantly lower than that of healthy controls (mean difference = −0.86, 95% CI [−1.67, −0.06], *p* = 0.033). Patients also scored significantly lower than healthy controls (mean difference = −1.00, 95% CI [−1.81, −0.20], *p* = 0.010). Finally, the difference between patients and their siblings was not statistically significant (mean difference = −0.14, 95% CI [−0.96, 0.68], *p* = 0.912). See Figure 1B for a boxplot depicting the behavioral results.

### 3.3 fMRI Analyses—Brain Activation

Unthresholded whole‐brain maps of within‐ and between‐group analyses are available on NeuroVault (Gorgolewski et al. 2015) (https://neurovault.org/collections/20556/).

#### 3.3.1 Within‐Group Analyses

Within each group, the one‐sample *t*‐test for Negative Faces > Control Condition identified significant activations across multiple brain regions commonly associated with emotional face processing, such as the bilateral amygdala, thalamus, and fusiform face areas. Several deactivations were also observed, particularly in regions of the Default Mode Network (DMN), comprising the bilateral ventromedial prefrontal cortex (vmPFC), precuneus, and posterior cingulate cortex. Decreased activations were also observed in the bilateral cuneus, somatosensory cortex, superior temporal gyrus, middle inferior temporal gyrus, and cerebellum during negative faces compared with the control condition.

**FIGURE 2:**
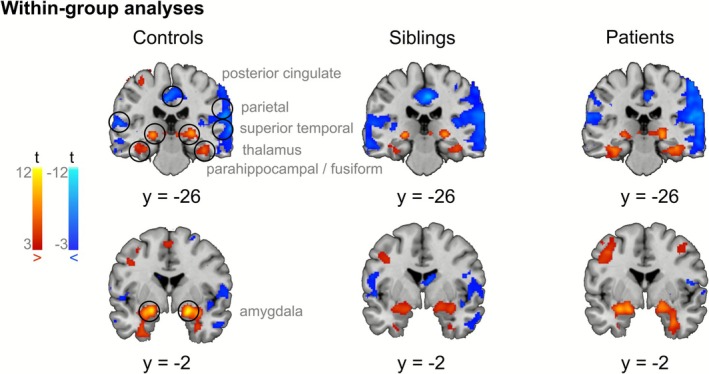
Results of within‐group brain activation analyses in healthy controls, siblings, and patients with schizophrenia. Brain regions showing significantly increased activations toward negative faces compared with the control condition are depicted in red, and deactivations are depicted in blue. As expected, the task activated regions involved in emotion processing, while deactivations were observed in regions of the DMN. Results are thresholded using a cluster‐forming threshold of *p*_voxel_ < 0.001 and a cluster significance threshold of *p*_cluster_ < 0.05.

The pattern of brain (de‐)activations was consistent across patients with schizophrenia, their siblings, and healthy controls (see Figure 2 for brain activation patterns in each group).

#### 3.3.2 Between‐Group Analyses

Using a cluster‐forming threshold *p*_voxel_ < 0.001 and FWE‐corrected *p*_cluster_ < 0.05, no significant group differences emerged.

**FIGURE 3:**
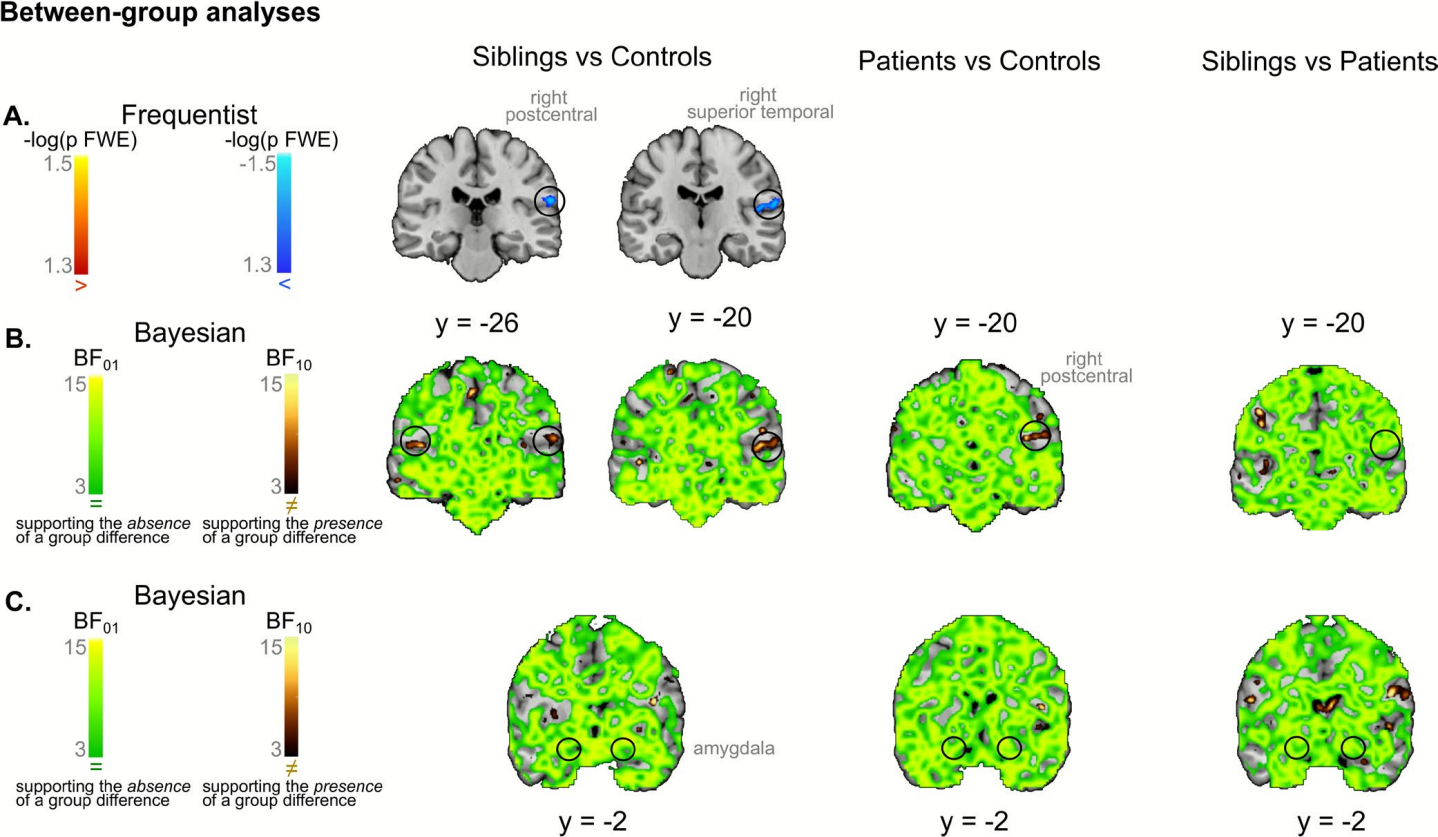
Results of between‐group two‐sample analyses. For frequentist analyses (A), hyperactivations are depicted in red, and hypoactivations in blue. Results are thresholded with a *p* < 0.05, FWE‐corrected, TFCE. For Bayesian analyses (B and C), voxels with at least moderate evidence supporting the absence of differences between groups (BF_01_ > 3) are depicted in green. Voxels with at least moderate evidence supporting the presence of a group difference (BF_10_ > 3) are depicted in gold. Results are thresholded at BF > 3. Bayesian analyses not only replicate the frequentist findings but also extend them by revealing a potential endophenotypic pattern in the right postcentral gyrus (B). Additionally, these analyses provide evidence for the absence of group differences in the amygdala (C). All functional maps are overlaid on the Colin 27 anatomical template.

Given the expectation of small effect sizes in siblings, a more liberal cluster‐forming threshold *p*_voxel_ < 0.005, still associated with an FWE‐corrected *p*_cluster_ < 0.05, was applied. This exploratory analysis revealed reduced activity in siblings as compared to healthy controls in a cluster encompassing the right superior temporal gyrus (MNI [*x y z*] [52, −20, 12], *T* = 3.53) and the right postcentral gyrus ([46, −6, 16], *T* = 4.01), as well as in the middle frontal gyrus ([−30, 38, 8], *T* = 4.49) (see Figure S1). As the results of this analysis were sensitive to the initial cluster‐forming threshold, we reproduced it using a Threshold‐Free Cluster Enhancement (TFCE) procedure with the default parameters (*E* = 0.5, *H* = 2; Smith and Nichols 2009) and 10.000 permutations. This procedure jointly considers voxel intensity and spatial extent to identify significant clusters without requiring an arbitrary cluster‐defining threshold, thus providing a threshold‐independent replication test. The analysis confirmed significant (*p* < 0.05, FWE‐corrected, TFCE) reduced activity in siblings as compared to healthy controls in a cluster encompassing the right superior temporal gyrus and right postcentral gyrus (see Figure 3A).

**FIGURE 4:**
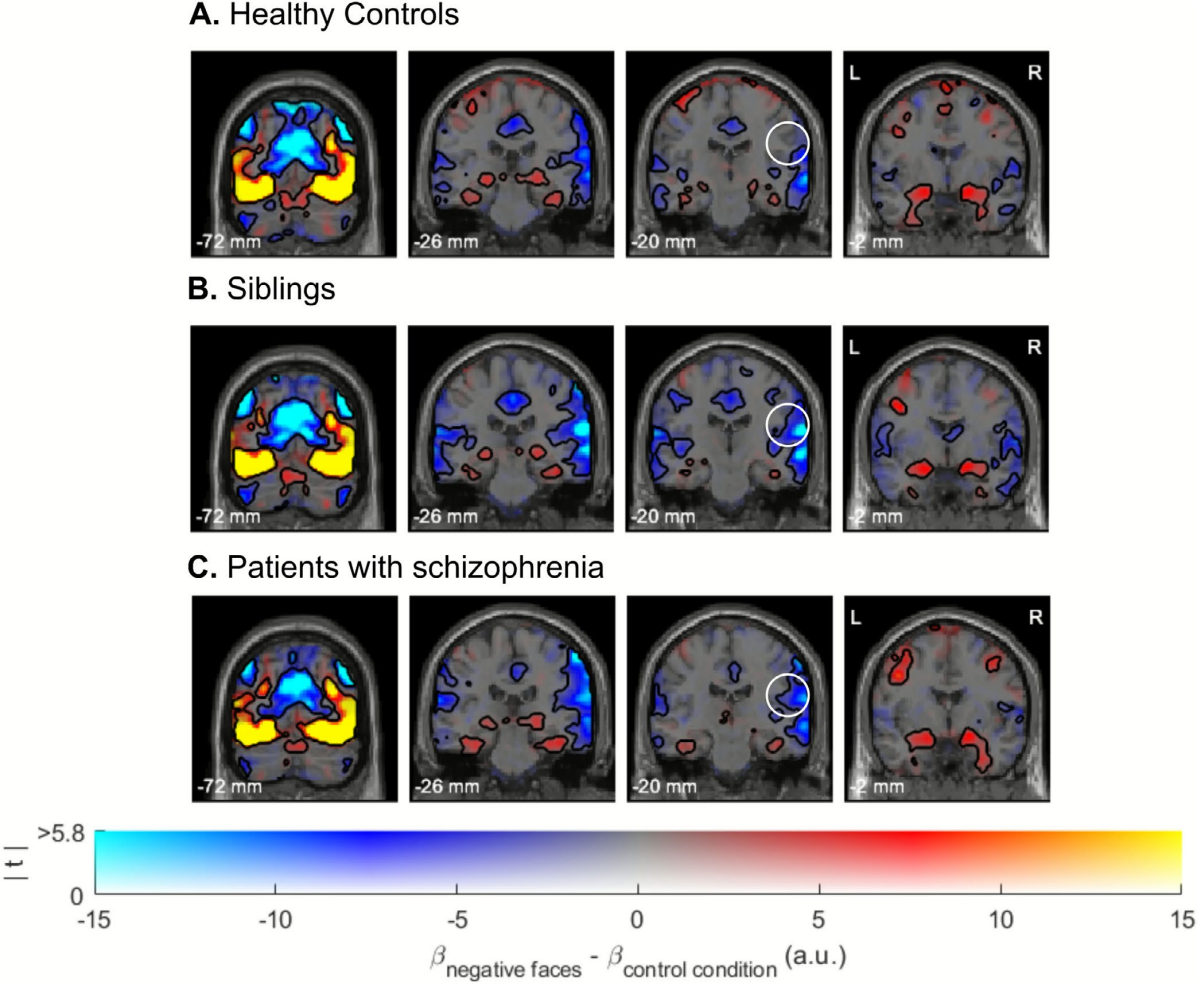
Results of within‐group brain activation analyses using dual‐coded images (Allen et al. 2012; Zandbelt 2017). In these images, color represents the contrast estimate—the difference between the beta values for negative faces and the beta values for the control condition, expressed in arbitrary units. Red regions indicate activations, while blue regions indicate deactivations. Transparency represents *t*‐values, which are computed by dividing the beta values by their estimated standard errors, thus accounting for the variability of the data. Black line contours denote significant (de‐)activations using a cluster‐forming threshold of *p*_voxel_ < 0.001 and a cluster significance threshold of *p*_cluster_ < 0.05. The white circle surrounding the cluster in the right superior temporal and postcentral gyrus highlights significant activation differences between siblings and healthy controls. All functional maps are overlaid on the SPM12 anatomical template.

For each group comparison, Bayesian analyses supported the absence of activation differences, with Bayes Factors (BF_01_) exceeding 3 across most voxels of the brain, including the bilateral amygdala (see Figure 3C). Several clusters exhibited at least moderate evidence of differences in brain activation between groups (BF_10_ > 3). These clusters were mostly in accordance with frequentist analyses. However, since Bayesian analyses use a less stringent procedure for multiple comparison corrections, which does not take into account cluster size, small clusters that are not in accordance with frequentist results are assumed to reflect false positives. It is noteworthy that, in addition to confirming significant differences between siblings and healthy controls in a right‐lateralized cluster comprising the superior temporal gyrus and the postcentral gyrus, Bayesian analyses also revealed evidence of group differences in a contralateral cluster in the left hemisphere. Additionally, Bayesian comparisons between patients and healthy controls revealed a cluster in the right postcentral gyrus where the Bayes Factors favored the alternative hypothesis (see Figure 3B). Note that this is a two‐tailed test, which does not indicate the direction of these differences; however, the directionality can be inferred by examining the unthresholded beta contrasts from the frequentist analyses (as depicted in dual‐coded Figure 4). As illustrated in this figure, Bayesian analyses provide evidence indicating a reduction in activation within this cluster in patients compared with healthy controls.

### 3.4 fMRI Analyses—Functional Connectivity

#### 3.4.1 Within‐Group Analyses

**FIGURE 5:**
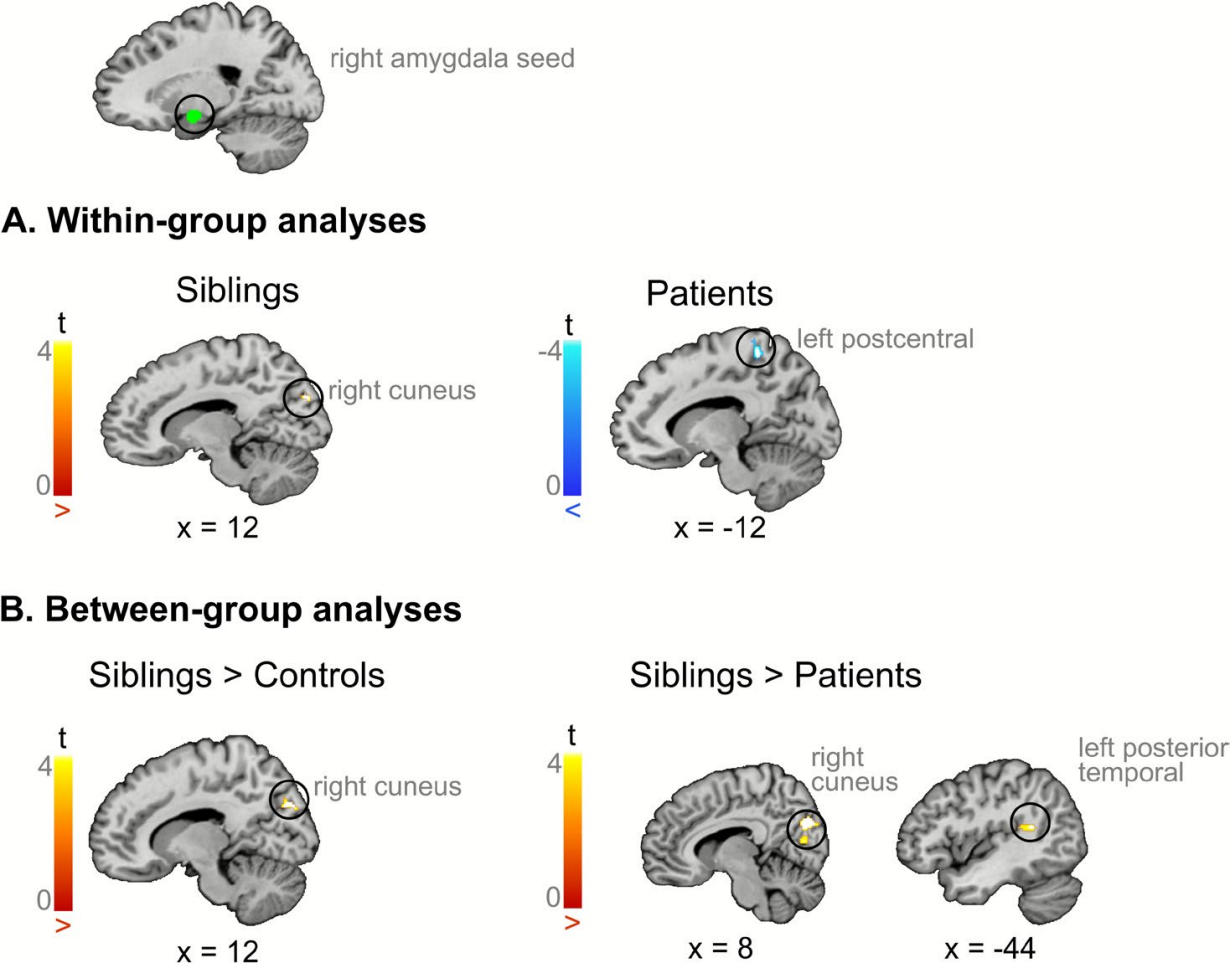
Results of connectivity analyses using the right amygdala as the seed of the PPI. For within‐group analyses (A), and between‐group analyses (B). Increased connectivity with the right amygdala is depicted in red, and decreased connectivity in blue. Siblings exhibited increased connectivity between the right amygdala and right cuneus, which was significantly greater than that observed in both controls and patients. Results are thresholded with a cluster‐forming threshold of *p*_voxel_ < 0.001 and a cluster significance threshold of *p*_cluster_ < 0.05. All functional maps are overlaid on the Colin 27 anatomical template.

gPPI analyses revealed that, when attending to negative faces as compared with the control condition, siblings showed increased connectivity between the right amygdala and right cuneus ([8, −78, 28], *T* = 5.00) (Figure 5A). In patients, there was a task‐dependent decrease in connectivity between the right amygdala and left postcentral gyrus ([−10, −42, 62], *T* = 5.43) (Figure 5A). No significant differences in connectivity were observed while using the left amygdala as the seed region. Furthermore, attending to negative faces versus the control condition was not associated with a significant change in functional connectivity in healthy controls.

#### 3.4.2 Between‐Group Analyses

**FIGURE 6:**
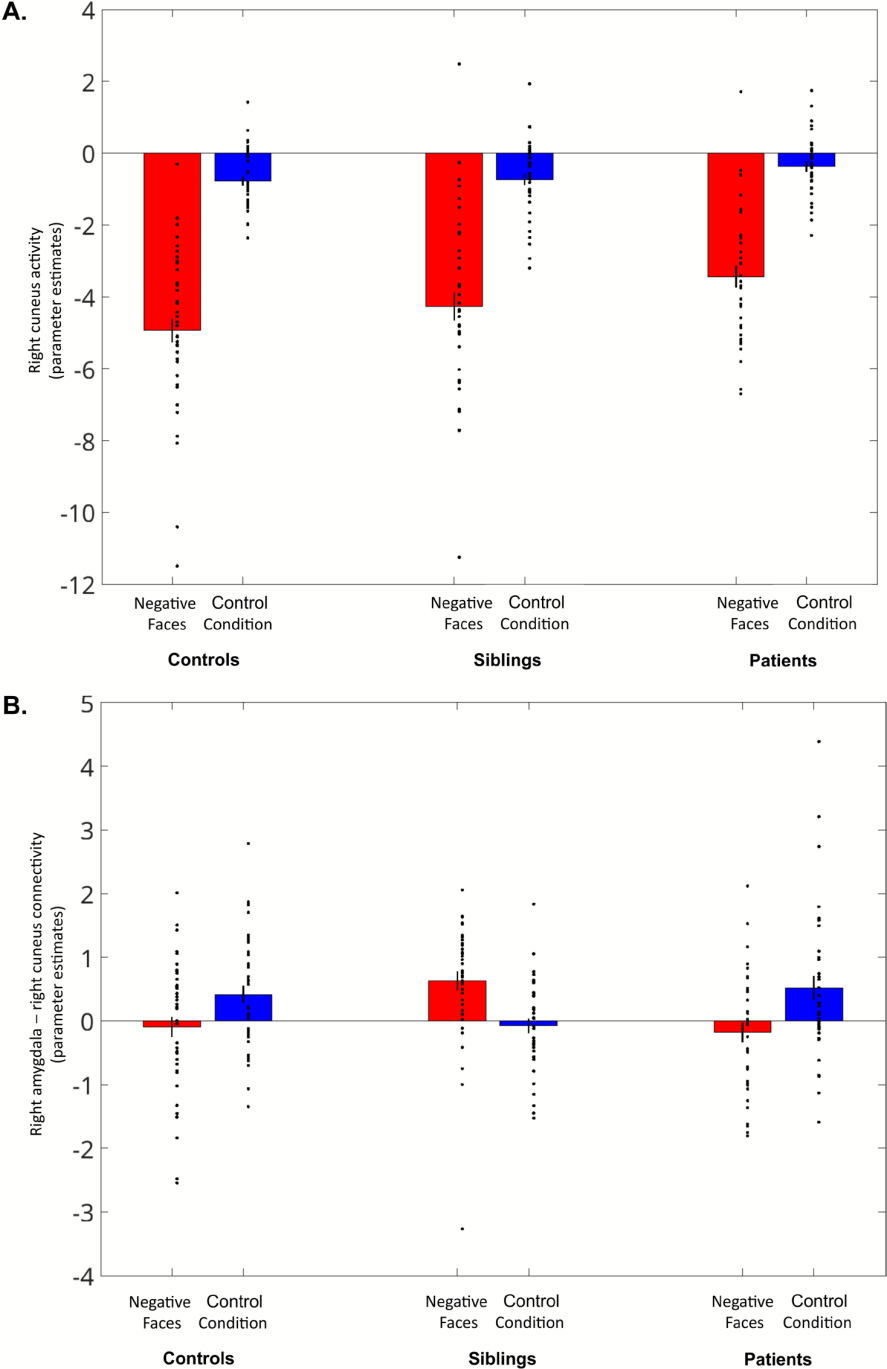
Barplot illustrating the modulation of the right cuneus activity (A) and of the task‐related connectivity between the right cuneus and the right amygdala (B) as a function of the condition (Negative Faces, Control Condition) and the group (controls, siblings, patients). The parameter estimates of the beta values were extracted from a mask of the right cuneus, defined as those voxels showing increased task‐related connectivity with the right amygdala in siblings as compared with healthy controls. While an endophenotypic pattern was observed in the activity of the right cuneus, siblings showed an opposite pattern of connectivity between the right cuneus and the right amygdala relative to both controls and patients. Individual data points are displayed, with error bars representing the standard error of the mean.

In response to negative faces versus the control condition, pairwise comparisons revealed that siblings exhibited stronger task‐related functional connectivity between the right amygdala and the right cuneus ([8, −80, 30], *T* = 4.61) compared with healthy controls (Figure 5B). When applying a more liberal cluster‐forming threshold (i.e., *p*_voxel_ < 0.005, FWE‐corrected *p*_cluster_ < 0.05) in exploratory analyses, this effect extended bilaterally within the cuneus (see Figure S2). We used the voxels in the right cuneus to create a mask, and subsequently extracted and plotted both activation and connectivity with the right amygdala across the three groups using *rfxplot* (Gläscher 2009) (see Figure 6). While activation in the right cuneus followed a qualitative endophenotypic pattern, with intermediate values in siblings relative to healthy controls and patients, the connectivity between the right cuneus and the right amygdala exhibited an opposite pattern in siblings compared with both healthy controls and patients.

Moreover, when siblings were compared with patients with schizophrenia, they showed increased connectivity between the right amygdala seed and various regions, including the right cuneus ([8, −80, 28], *T* = 5.31) extending into the right lateral reminder of the occipital lobe ([28, −66, 20], *T* = 4.94), left posterior temporal lobe ([−48, −44, 14], *T* = 5.33), and left cerebellum ([−10, −68, −8], *T* = 4.69). (Figure 5B). No significant differences between groups in functional connectivity were observed when comparing patients with schizophrenia and healthy controls.

## 4 Discussion

The current study aimed to investigate the neural mechanisms underlying the perception of negative emotional faces in patients with schizophrenia, their healthy first‐degree relatives (siblings), and healthy controls. Our findings provide evidence that siblings of patients with schizophrenia exhibit both behavioral and neural impairments compared with healthy controls. This supports the growing body of literature suggesting that such impairments may already be present in individuals at genetic risk for schizophrenia, indicating a potential genetic component underlying these deficits.

Using an ecological emotional task, we observed that both patients with schizophrenia and their siblings exhibited poorer performance in recognizing negative facial expressions, as reflected by lower BIS. Importantly, by integrating both reaction times and accuracy, the BIS provides a more accurate reflection of overall task efficiency. This finding is consistent with previous research showing that patients with schizophrenia have impaired emotion recognition, particularly for negatively valenced expressions (Chan et al. 2010; Gao et al. 2021; Kohler et al. 2003). Although both patients and siblings performed worse than healthy controls, no significant difference was observed between these two groups. However, as qualitatively illustrated in the bar plot (see Figure 1), siblings exhibited slightly milder deficits. These results align with earlier meta‐analytic findings showing that first‐degree relatives of individuals with schizophrenia exhibit difficulties in recognizing facial expressions, particularly negative emotions (Martin et al. 2020).

At the neural level, our whole‐brain activation analysis demonstrated that patients, siblings, and healthy controls all engaged regions commonly associated with emotion processing, including the bilateral amygdala, thalamus, and fusiform face areas. This finding is consistent with our expectations, as the emotional task was specifically designed to robustly engage brain circuits involved in emotion processing, as demonstrated in prior fMRI studies (Comte et al. 2016). We also observed greater engagement of the DMN during the control condition, which required less cognitive effort, compared with the negative faces. This aligns with previous research indicating increased involvement of the DMN with decreasing levels of cognitive effort (Weber et al. 2022).

When comparing brain activations between siblings and healthy controls, exploratory analyses using a more liberal cluster‐forming threshold (i.e., *p*_voxel_ < 0.005, FWE‐corrected *p*_cluster_ < 0.05) revealed reduced activation in a cluster comprising the right superior temporal gyrus and the right postcentral gyrus in siblings. Although these results should be interpreted with caution due to the liberal threshold, they were supported by complementary analyses. Specifically, TFCE and Bayesian analyses both confirmed reduced activity in this region, with Bayesian results additionally suggesting bilateral hypoactivation in this region among siblings compared with healthy controls. Together, these findings suggest that siblings may exhibit subtle but detectable alterations in neural responses to negative emotional faces, even in the absence of a clinical diagnosis.

The superior temporal gyrus has been strongly implicated in the pathogenesis of schizophrenia. A recent meta‐analysis of 246 MRI studies revealed that patients with schizophrenia exhibit volume reductions in this region (Kuo and Pogue‐Geile 2019). Additionally, reduced cortical connectivity with the superior temporal gyrus has also been observed in both patients and their relatives (Oertel‐Knöchel et al. 2013). Finally, evidence for the involvement of this region in the transition to schizophrenia comes from studies reporting gray matter reduction in this region in both first‐episode psychosis and ultrahigh‐risk individuals who subsequently developed psychosis at 12‐month follow‐up, as compared with those who did not (Takahashi et al. 2009).

Previous studies have also frequently reported structural and functional deficits in the postcentral gyrus, which encompasses the somatosensory cortex and corresponds to the cluster identified in this study. Notably, decreased gray matter volume has been observed in chronic patients, particularly in those with adolescent‐onset schizophrenia (Zhang et al. 2017), as well as in individuals with first‐episode psychosis (Job et al. 2002), with further reductions at one‐year follow‐up (Ferro et al. 2015). Moreover, there is substantial evidence of hypoconnectivity within the somatosensory network (Dong, Wang, Chang, et al. 2018). Alterations of its connection to the thalamus have also been observed (Kaufmann et al. 2015; Li et al. 2017). Interestingly, this region has been associated with emotion recognition performances in schizophrenia. Indeed, a 10‐week emotion recognition training in patients with schizophrenia demonstrated increased activation in the right postcentral gyrus, which could predict behavioral improvements (Hooker et al. 2012).

Reduced activation in the right superior temporal and postcentral gyri, observed in siblings of patients, may have significant implications for emotion perception. This finding aligns with the right hemisphere hypothesis, which suggests that emotional processing and related behaviors are predominantly lateralized to the right hemisphere (Demaree et al. 2005). Additionally, these regions are involved in the theory of mind (Bodden et al. 2013; Caillaud et al. 2020; Mier et al. 2010) and the embodiment of emotions (Barrett 2017; Kropf et al. 2019). Emotion recognition is closely related to these functions, as accurately identifying others' emotions often requires the ability to adopt their perspective and involves the simulation of their affective states via somatosensory representation (Adolphs et al. 2000; Mitchell and Phillips 2015). While remaining cautious with reverse inference and post hoc interpretation, we suggest that reduced involvement of the cluster encompassing the right superior temporal and the right postcentral gyrus in siblings of patients with schizophrenia may indicate diminished engagement in higher‐level cognitive processes essential for social cognition.

Furthermore, Bayesian analyses provide evidence in favor of the absence of differences between siblings and controls across most of the voxels of the brain, and particularly in the amygdala (see Figure 3C). This is in line with our previous meta‐analysis showing no reliable differences between individuals at enhanced risk of schizophrenia and healthy controls, with strong evidence in the bilateral amygdala (Fiorito et al. 2022).

We did not observe significant brain activation differences between patients and healthy controls, with evidence in favor of the absence of group differences across most voxels of the brain comprising the bilateral amygdala (see Figure 3C). This contrasts with previous meta‐analytical findings reporting decreased activations, notably in limbic regions, in schizophrenia (Dong, Wang, Jia, et al. 2018; Li et al. 2010; Taylor et al. 2012). However, our findings are consistent with the hypothesis that the hypoactivations observed in patients may have been, at least partially, overestimated due to the use of neutral stimuli as a comparator in fMRI studies (Anticevic et al. 2012; Dugré et al. 2019; Fiorito et al. 2024).

It is important to note that Bayesian analyses revealed at least moderate evidence of differences between patients with schizophrenia and healthy controls within a cluster in the right postcentral gyrus. This finding may suggest a shared pathway affecting both patients and their siblings, indicating impairments in higher‐level emotional processing, rather than implicating amygdala dysfunction as traditionally hypothesized when using neutral stimuli as comparators. We thus suggest that the observed reductions could indicate a potential endophenotypic marker. However, this result should be interpreted with caution due to its lack of replication in classical frequentist analyses. The absence of significant differences might be attributed to substantial variability among patients (as observed in dual‐color Figure 4). Schizophrenia is an exceptionally heterogeneous disorder with diverse clinical manifestations, biological predispositions, and environmental influences. Stratifying patients into subgroups with distinct pathophysiological and clinical profiles could potentially enhance our understanding of the underlying mechanisms and provide deeper insights into the complexity of the disorder. Such an approach may also help in identifying and validating more robust endophenotypes.

Functional connectivity analyses using gPPI revealed notable differences in siblings compared with healthy controls, showing increased task‐related connectivity between the right amygdala and right cuneus. This result reinforces the notion that traditional activation‐based analyses may not fully capture the neural abnormalities associated with emotion processing in individuals at risk of schizophrenia.

The involvement of the right amygdala in this pattern is consistent with prior findings suggesting that this region is particularly responsive to image‐related affective information, in contrast to the left amygdala, which may be more engaged in language‐related affective processing (Markowitsch 1998).

The cuneus is a region of the visual cortex that receives visual input from the contralateral superior retina. Research indicates that emotion and stress may influence its function. Indeed, EEG studies have reported that the C1 component, which primarily originates in the cuneus, is enhanced in response to fearful faces but not neutral or happy faces, thus showing that negative emotions may modulate the early stages of visual processing via their impact on the cuneus (Raftopoulos 2023). Additionally, previous research supports a dual‐route model for processing negative facial expressions, as proposed by LeDoux (1996). This model suggests that only the processing of negative emotions, unlike positive ones, activates both cortical (right cuneus to right amygdala) and subcortical (right thalamus to right amygdala) pathways (Liu et al. 2015). Following acute stress, reduced connectivity between the amygdala and the cuneus has been observed (Ford et al. 2022; Quaedflieg et al. 2015), while recovery from stress has been associated with increased functional connectivity between the amygdala and the cuneus (Quaedflieg et al. 2015). Additionally, elevated peripheral inflammatory markers have been linked to reduced amygdala‐cuneus connectivity during negative face recognition (Swartz et al. 2022). Beyond its role in primary visual processing, the cuneus appears to contribute to working memory (Bomyea et al. 2017; He et al. 2021; Owens et al. 2018), and to the regulation of emotions associated with negative autobiographical memories (Kross et al. 2009).

Clinically, the cuneus has been previously implicated in schizophrenia pathogenesis, based on findings showing decreased gray matter volume (Mané et al. 2009; Yu et al. 2018), impaired dynamic functional connectivity at rest (Nyatega et al. 2021), and decreased activations during retrieval of episodic memory—the part of the memory more closely linked to the emotional component (Ragland et al. 2009). Recent meta‐analytical results also showed decreased activations in the cuneus during theory of mind tasks in patients as compared with healthy controls (Jáni and Kašpárek 2018; Weng et al. 2022). Reduced activity in the cuneus has also been reported in previous studies investigating emotional faces as compared with scrambled ones in healthy first‐degree relatives of patients with schizophrenia (Spilka et al. 2015).

Few studies have investigated brain connectivity in first‐degree relatives of patients with schizophrenia. One study using dynamic causal modeling (DCM) reported reduced driving inputs to the V1 area in the offspring of patients with schizophrenia during an emotional n‐back paradigm involving visual faces (Diwadkar et al. 2012). This finding could not be replicated in a subsequent study, since the authors did not include visual areas in their model (Quarto et al. 2018). However, similar results were reported in another study that utilized correlation‐based network analyses to investigate functional connectivity (Cao et al. 2016). In this study, they observed decreased coupling in first‐degree relatives among several generations (but mostly including offspring) in several nodes of the limbic system, including the amygdala and visual cortex areas such as the cuneus, during emotional face processing (Cao et al. 2016).

When extracting both activation and connectivity estimates from the right cuneus, we observed an endophenotypic pattern for activation, with intermediate activation estimates in siblings and an opposite pattern of connectivity with the right amygdala in siblings compared with healthy controls and patients. Based on this observation and in light of previous literature indicating reduced connectivity between the amygdala and cuneus during negative face processing in the offspring of patients with schizophrenia (Cao et al. 2016; Diwadkar et al. 2012), we speculate that the increased connectivity between the right amygdala and the right cuneus may represent a compensatory mechanism in low‐level areas of face emotion processing in siblings of patients with schizophrenia. Notably, previous studies primarily investigated brain connectivity in children of patients with schizophrenia, while our study included siblings who are older and thus considered less at risk due to their age. Interestingly, enhanced recruitment of the cuneus has recently been proposed as a functional compensation mechanism, particularly in healthy aging, which may facilitate the processing of multiple features of a stimulus (Knights et al. 2024). Furthermore, it has been suggested that genetic resilience in schizophrenia may emerge through the strengthening of visual pathways (Hettwer et al. 2022).

We did not observe brain connectivity differences between patients with schizophrenia and healthy controls. Previous research suggests that antipsychotic drugs may lead to network topologies that are closer to those of healthy subjects (Towlson et al. 2019). When comparing siblings with patients with schizophrenia, we observed reduced connectivity in patients between the right amygdala and right cuneus extending in the lateral reminder of the occipital lobe, the left posterior temporal lobe, and the left cerebellum. It has been shown that individuals with cannabis use disorder have decreased functional connectivity between the amygdala and the cuneus (Aloi et al. 2021). Since 26% of patients with schizophrenia present a comorbid cannabis use disorder (Hunt et al. 2018), and 42% of patients have a lifetime use of cannabis (Green et al. 2005), cannabis consumption may have impacted the observed results.

This study has several limitations. First, the sample size is relatively small. Nevertheless, a key strength of this study is the inclusion of both patients and siblings from the same families, in contrast to previous research that has often examined these groups separately. This approach helps mitigate the influence of environmental factors, as it controls for shared familial and environmental variables that could otherwise confound the results. Large‐scale studies should replicate these findings. The inclusion of a large number of participants should also allow the investigation of brain‐behavior correlations. Indeed, it has been suggested that such associations between brain functioning and complex cognitive phenotypes require samples with thousands of individuals in order to be reliable (Marek et al. 2022). Second, the groups included in this study were not comparable in terms of sex and education level, which may have introduced a bias in our results. Third, all patients were undergoing pharmacological treatment. Although we included only those who had been stable (with no changes in treatment or dosage for at least six months), it is acknowledged that antipsychotics can affect brain structure and function. Future research should investigate emotional brain correlates in individuals with first‐episode psychosis who have not yet received pharmacological treatment. Fourth, siblings and controls were free of psychiatric diagnoses, which may have introduced further clinical differences with patients beyond the diagnosis of schizophrenia, since patients often present comorbidities. Still, we chose this approach because excluding patients with comorbidities would have greatly limited generalizability, while including affected siblings would have confounded our ability to isolate genetic vulnerability to schizophrenia. Future studies should adopt dimensional approaches to capture a wider range of symptom variability across groups. Finally, in this study, we were unable to clearly differentiate between risk factors, protective factors, or compensatory mechanisms. Future longitudinal studies that follow relatives who transition to schizophrenia and those who do not will be crucial.

## Author Contributions

Concept and design: F.C.S., E.F. Acquisition: A.K., C.M.‐P., N.O., M.R., F.C.S., E.F. Analysis and interpretation of data: A.M.F., G.S., E.F. Statistical analysis: A.M.F., H.H., G.S., E.F. Drafting of the manuscript: A.M.F., G.S., E.F. Obtained funding: E.F.

## Funding

A.M.F. was supported by the Fondation Pierre Deniker, Fondation Neurodis, and Hospital Center Le Vinatier. G.S. was supported by the Agence Nationale de la Recherche (Grant number ANR‐19‐ce37‐0012‐01) and the Fondation NRJ‐Institut de France. E.F. was supported by the Fondation de France and University Hospital of Saint‐Etienne. The funding source had no role in the design and conduct of the study; collection, management, analysis, and interpretation of the data; preparation, review, or approval of the manuscript; and decision to submit the manuscript for publication.

## Ethics Statement

This study received the ethical authorization of the French *Comité de Protection des Personnes*.

## Consent

Participants were informed about the study and procedures and signed an informed consent before taking part in the study.

## Conflicts of Interest

E.F. has received consultancy fees from Bristol Myers Squibb, Janssen‐Cilag, Lilly, Lundbeck, Otsuka Pharmaceutical Co. Ltd., Recordatti, and Sanofi and has lectured for AbbVie, AstraZeneca, Bristol Myers Squibb, Janssen‐Cilag, Lundbeck, Otsuka, MSD, and Sanofi. All other authors report no biomedical financial interest or potential conflicts of interest.

## Supporting information


**Data S1:** hbm70437‐sup‐0001‐Figures.pdf.

## Data Availability

A.M.F. and G.S have full access to all the data in the study and take responsibility for the integrity of the data and the accuracy of the data analysis. The data that support the findings of this study are available from A.M.F and G.S upon reasonable request. Unthresholded whole‐brain maps of within‐ and between‐group analyses are available on NeuroVault (https://neurovault.org/collections/20556/).
