# Supplementary material for: Emotion Processing in Schizophrenia: Insights From a Brain Imaging Study Comparing Patients, Siblings, and Healthy Controls
Source: Hum Brain Mapp. 2026 Feb 18;47(3):e70437. doi: 10.1002/hbm.70437 (PMC12916247; doi:10.1002/hbm.70437)
Supplement: Supplementary file 1 — Data S1: hbm70437‐sup‐0001‐Figures.pdf. [file HBM-47-e70437-s001.pdf]

SUPPLEMENTARY MATERIAL

Emotion processing in schizophrenia: insights from a brain imaging study comparing patients, siblings, and healthy controls

Fiorito *et al.*

Contents:

Supplementary Figure 1. *Results of exploratory analyses of activation in siblings versus healthy controls.* 2

Supplementary Figure 2. *Results of exploratory analyses of connectivity in siblings versus healthy controls.* 3

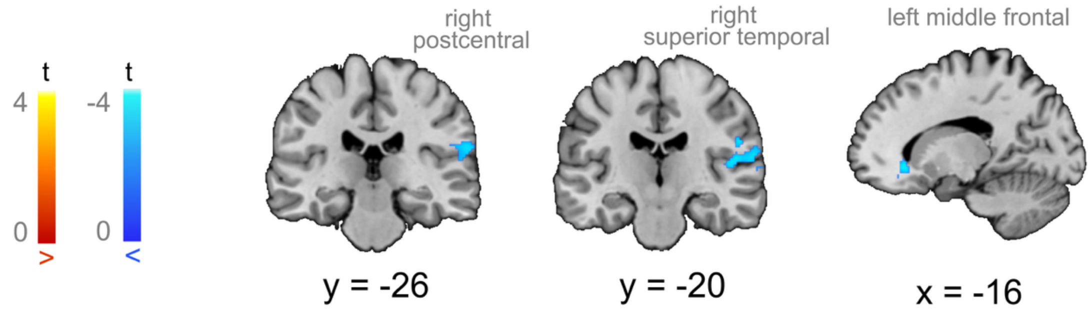

Supplementary Figure 1. Results of exploratory analyses of activation in siblings versus healthy controls. *Hyperactivations are depicted in red, and hypoactivations in blue. Results are thresholded with* $p_{voxel} < 0.005$ *and FWE-corrected* $p_{cluster} < 0.05$*. All functional maps are overlaid on the Colin 27 anatomical template.*

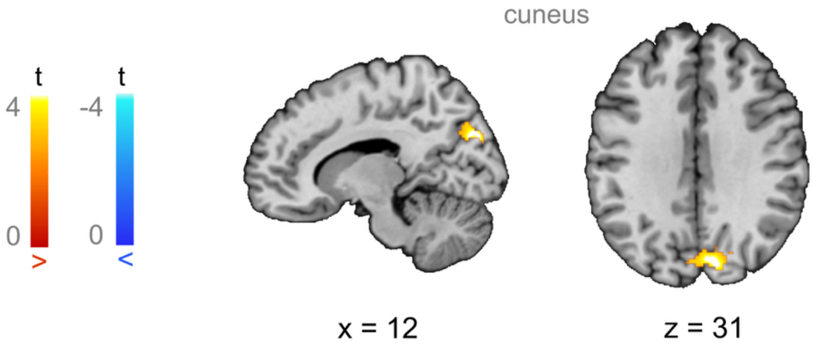

Supplementary Figure 2. Results of exploratory analyses of connectivity in siblings versus healthy controls. *Hyperactivations are depicted in red, and hypoactivations in blue. Results are thresholded with* $p_{voxel} < 0.005$ *and FWE-corrected* $p_{cluster} < 0.05$*. All functional maps are overlaid on the Colin 27 anatomical template.*
